# Bis(thio­semicarbazide)nickel(II) bis­[2-(thio­semicarbazonometh­yl)benzene­sulfonate] dihydrate

**DOI:** 10.1107/S1600536809022144

**Published:** 2009-06-13

**Authors:** Wei Zhang, Yuan-Tao Chen

**Affiliations:** a Department of Chemistry, Qinghai Normal University, Xining 810008, People’s Republic of China

## Abstract

In the title compound, [Ni(CH_5_N_3_S)_2_](C_8_H_8_N_3_O_3_S_2_)_2_·2H_2_O, the Ni^II^ atom lies on a inversion centre and is four-coordinated by two N and two S atoms of two thio­semicarbazide ligands in an almost square-planar coordination. In the crystal structure, the molecules are linked into a three-dimensional network *via* C—H⋯O, C—H⋯N, N—H⋯O, N—H⋯S and O—H⋯O hydrogen bonds.

## Related literature

For the design and synthesis of organic–inorganic hybrid materials and their potential practical applications, see: Hagrman *et al.* (1998 ▶); Ranford *et al.* (1998 ▶). 
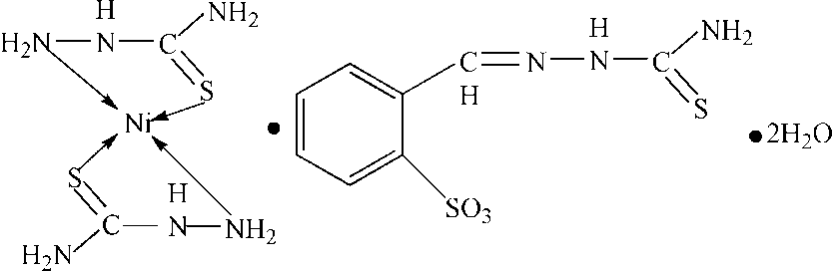


## Experimental

### 

#### Crystal data

[Ni(CH_5_N_3_S)_2_](C_8_H_8_N_3_O_3_S_2_)_2_·2H_2_O
*M* *_r_* = 793.61
Triclinic, 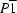

*a* = 7.3853 (8) Å
*b* = 9.9043 (11) Å
*c* = 11.3140 (18) Å
α = 86.670 (2)°
β = 77.611 (1)°
γ = 75.177 (1)°
*V* = 781.40 (17) Å^3^
*Z* = 1
Mo *K*α radiation
μ = 1.09 mm^−1^
*T* = 298 K
0.33 × 0.21 × 0.13 mm

#### Data collection

Bruker SMART CCD area-detector diffractometer
Absorption correction: multi-scan (*SADABS*; Sheldrick, 1996 ▶) *T*_min_ = 0.716, *T*_max_ = 0.872
4091 measured reflections
2717 independent reflections
2268 reflections with *I* > 2σ(*I*)
*R*_int_ = 0.014

#### Refinement

*R*[*F* ^2^ > 2σ(*F* ^2^)] = 0.029
*wR*(*F* ^2^) = 0.078
*S* = 1.03
2717 reflections
205 parameters
H-atom parameters constrained
Δρ_max_ = 0.30 e Å^−3^
Δρ_min_ = −0.25 e Å^−3^

### 

Data collection: *SMART* (Bruker, 2000 ▶); cell refinement: *SAINT* (Bruker, 2000 ▶); data reduction: *SAINT*; program(s) used to solve structure: *SHELXS97* (Sheldrick, 2008 ▶); program(s) used to refine structure: *SHELXL97* (Sheldrick, 2008 ▶); molecular graphics: *SHELXTL* (Sheldrick, 2008 ▶); software used to prepare material for publication: *SHELXTL*.

## Supplementary Material

Crystal structure: contains datablocks global, I. DOI: 10.1107/S1600536809022144/at2808sup1.cif

Structure factors: contains datablocks I. DOI: 10.1107/S1600536809022144/at2808Isup2.hkl

Additional supplementary materials:  crystallographic information; 3D view; checkCIF report

## Figures and Tables

**Table:** 

Ni1—N5	1.903 (2)
Ni1—S3	2.1788 (7)

**Table:** 

N5^i^—Ni1—N5	180
N5^i^—Ni1—S3	91.59 (6)
N5—Ni1—S3	88.41 (6)

Symmetry code: (i) 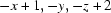.

**Table 2:** Hydrogen-bond geometry (Å, °)

*D*—H⋯*A*	*D*—H	H⋯*A*	*D*⋯*A*	*D*—H⋯*A*
N1—H1⋯S2^ii^	0.86	2.73	3.475 (2)	147
N3—H3*A*⋯N2	0.86	2.29	2.643 (3)	105
N3—H3*A*⋯O4^iii^	0.86	2.58	3.224 (3)	132
N4—H4⋯O4^iii^	0.86	1.97	2.794 (3)	160
O4—H4*C*⋯O2	0.85	1.97	2.819 (3)	172
O4—H4*D*⋯O3^iv^	0.85	2.19	3.036 (3)	172
O4—H4*D*⋯O4^v^	0.85	2.58	2.903 (3)	104
N5—H5*A*⋯O2^vi^	0.90	1.97	2.837 (3)	163
N5—H5*B*⋯O1^iii^	0.90	2.23	2.893 (3)	130
N6—H6*A*⋯O3^vii^	0.86	2.03	2.866 (3)	165
N6—H6*B*⋯S2^viii^	0.86	2.50	3.298 (2)	156
C2—H2⋯O1	0.93	2.50	3.066 (3)	119
C5—H5⋯O2	0.93	2.38	2.811 (3)	108
C8—H8⋯N2	0.93	2.48	2.790 (4)	100

Symmetry codes: (ii) 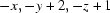; (iii) 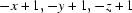; (iv) 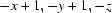; (v) 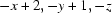; (vi) 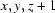; (vii) 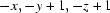; (viii) 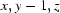.

**Fig. 1.**
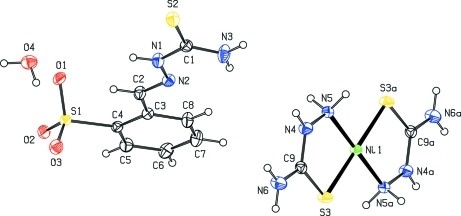
The molecular structure of (I) showing 30% displacement ellipsoids.

## supplementary crystallographic information

### Comment

The design and synthesis of organic/inorganic hybrid materials have attracted intense attention in recent years owing to their potential practical applications, such as antitumor, antidiabetic, antitubercular activities, magnetism and catalysis (Ranford, *et al.*, 1998; Hagrman, *et al.*, 1998). In order to achieve supramolecular transition metal complexes by self-assembly, and to explore the relationship between the structure and the biological properties, as one part of our systematic work, in this paper, we report on the synthesis and crystal structure of the title compound, (I).

As shown in Fig. 1, the Ni^II^ atom lies on a inversion centre and it is four-coordinate with two N donors and two S donors of two thiosemicarbazide ligands, and adopts distorted square coordination. The bond distances of Ni1—N5 (N5A) [1.903 (2) Å], Ni1—S3 (S3A) [2.1788 (7)Å] are consistent with the bond lengths reported previously. The bond distances of Ni1—N5 (N5A) are shorter than the Ni1—S3 (S3A), showing that the strength of Ni1—N5 (N5A) are stronger than the Ni1—S3(S3A) (Table 1). In the crystal packing, the molecules form a one-dimensional chain structure by the C—H···O, N—H···O, N—H···S and O—H···O hydrogen bonds (Table 2).

### Experimental

The solution of 1.0 mmol 2-formyl-benzenesulfonate-thiosemicarbazide was added to a solution of 0.5 mmol Ni(NCS)_2_.4H_2_O in 5 ml ethanol at room temperature. The mixture was refluxed for 4 h with stirring, then the resulting precipitate was filtered, washed, and dried *in vacuo* over P_4_O_10_ for 48 h. Single crystals suitable for X-ray structural analysis was obtained by slowly evaporating from methanol at room temperature.

### Refinement

All H atoms were placed geometrically and treated as riding on their parent atoms with O—H = 0.85 Å, C—H = 0.93 Å, N—H = 0.86-0.90 Å, and with U_iso_(H) = 1.2 U_eq_(C, N) or 1.5 U_eq_(O).

### Crystal data

**Table:**

[Ni(CH_5_N_3_S)_2_](C_8_H_8_N_3_O_3_S_2_)_2_·2H_2_O	*Z* = 1
*M**_r_* = 793.61	*F*(000) = 410
Triclinic, *P*1	*D*_x_ = 1.686 Mg m^−^^3^
Hall symbol: -P 1	Mo *K*α radiation, λ = 0.71073 Å
*a* = 7.3853 (8) Å	Cell parameters from 2430 reflections
*b* = 9.9043 (11) Å	θ = 2.8–28.3°
*c* = 11.3140 (18) Å	µ = 1.09 mm^−^^1^
α = 86.670 (2)°	*T* = 298 K
β = 77.611 (1)°	Block, light green
γ = 75.177 (1)°	0.33 × 0.21 × 0.13 mm
*V* = 781.40 (17) Å^3^	

### Data collection

**Table:**

Bruker SMART CCD area-detector diffractometer	2717 independent reflections
Radiation source: fine-focus sealed tube	2268 reflections with *I* > 2σ(*I*)
graphite	*R*_int_ = 0.014
φ and ω scans	θ_max_ = 25.0°, θ_min_ = 1.8°
Absorption correction: multi-scan (*SADABS*; Sheldrick, 1996)	*h* = −8→6
*T*_min_ = 0.716, *T*_max_ = 0.872	*k* = −11→10
4091 measured reflections	*l* = −13→13

### Refinement

**Table:**

Refinement on *F*^2^	Primary atom site location: structure-invariant direct methods
Least-squares matrix: full	Secondary atom site location: difference Fourier map
*R*[*F*^2^ > 2σ(*F*^2^)] = 0.029	Hydrogen site location: inferred from neighbouring sites
*wR*(*F*^2^) = 0.078	H-atom parameters constrained
*S* = 1.03	*w* = 1/[σ^2^(*F*_o_^2^) + (0.0372*P*)^2^ + 0.4266*P*] where *P* = (*F*_o_^2^ + 2*F*_c_^2^)/3
2717 reflections	(Δ/σ)_max_ < 0.001
205 parameters	Δρ_max_ = 0.30 e Å^−^^3^
0 restraints	Δρ_min_ = −0.25 e Å^−^^3^

### Special details

**Table:**

Geometry. All e.s.d.'s (except the e.s.d. in the dihedral angle between two l.s. planes) are estimated using the full covariance matrix. The cell e.s.d.'s are taken into account individually in the estimation of e.s.d.'s in distances, angles and torsion angles; correlations between e.s.d.'s in cell parameters are only used when they are defined by crystal symmetry. An approximate (isotropic) treatment of cell e.s.d.'s is used for estimating e.s.d.'s involving l.s. planes.
Refinement. Refinement of *F*^2^ against ALL reflections. The weighted *R*-factor *wR* and goodness of fit *S* are based on *F*^2^, conventional *R*-factors *R* are based on *F*, with *F* set to zero for negative *F*^2^. The threshold expression of *F*^2^ > σ(*F*^2^) is used only for calculating *R*-factors(gt) *etc*. and is not relevant to the choice of reflections for refinement. *R*-factors based on *F*^2^ are statistically about twice as large as those based on *F*, and *R*- factors based on ALL data will be even larger.

### Fractional atomic coordinates and isotropic or equivalent isotropic displacement parameters (Å^2^)

**Table:**

	*x*	*y*	*z*	*U*_iso_*/*U*_eq_	
Ni1	0.5000	0.0000	1.0000	0.02441 (13)	
S1	0.34631 (9)	0.48280 (6)	0.21469 (5)	0.02605 (16)	
S2	−0.17224 (10)	1.04164 (7)	0.68768 (6)	0.03721 (19)	
S3	0.26167 (9)	−0.01235 (7)	0.91972 (6)	0.03314 (17)	
N1	0.0111 (3)	0.8006 (2)	0.58317 (19)	0.0340 (5)	
H1	−0.0018	0.8437	0.5161	0.041*	
N2	0.1084 (3)	0.6619 (2)	0.58171 (19)	0.0303 (5)	
N3	−0.0510 (4)	0.7954 (2)	0.7884 (2)	0.0484 (7)	
H3A	0.0034	0.7075	0.7846	0.058*	
H3B	−0.0972	0.8354	0.8577	0.058*	
N4	0.2869 (3)	0.2479 (2)	0.9094 (2)	0.0334 (5)	
H4	0.2478	0.3364	0.8989	0.040*	
N5	0.4569 (3)	0.1917 (2)	0.95422 (19)	0.0290 (5)	
H5A	0.4527	0.2420	1.0189	0.035*	
H5B	0.5575	0.2025	0.8969	0.035*	
N6	0.0401 (3)	0.2105 (2)	0.8361 (2)	0.0408 (6)	
H6A	0.0047	0.2983	0.8208	0.049*	
H6B	−0.0232	0.1547	0.8197	0.049*	
O1	0.4008 (3)	0.61284 (18)	0.21793 (16)	0.0365 (4)	
O2	0.4767 (3)	0.38730 (18)	0.12150 (15)	0.0338 (4)	
O3	0.1474 (3)	0.50454 (19)	0.20417 (17)	0.0375 (5)	
O4	0.8292 (3)	0.46182 (19)	0.06672 (18)	0.0421 (5)	
H4C	0.7240	0.4388	0.0908	0.051*	
H4D	0.8465	0.4734	−0.0095	0.051*	
C1	−0.0636 (4)	0.8689 (3)	0.6882 (2)	0.0302 (6)	
C2	0.1743 (4)	0.6065 (3)	0.4783 (2)	0.0317 (6)	
H2	0.1525	0.6586	0.4094	0.038*	
C3	0.2855 (3)	0.4598 (3)	0.4667 (2)	0.0270 (5)	
C4	0.3691 (3)	0.3944 (2)	0.3547 (2)	0.0251 (5)	
C5	0.4773 (4)	0.2568 (3)	0.3488 (2)	0.0345 (6)	
H5	0.5331	0.2144	0.2740	0.041*	
C6	0.5023 (4)	0.1825 (3)	0.4542 (3)	0.0399 (7)	
H6	0.5753	0.0906	0.4501	0.048*	
C7	0.4188 (4)	0.2450 (3)	0.5650 (3)	0.0421 (7)	
H7	0.4347	0.1947	0.6357	0.051*	
C8	0.3117 (4)	0.3820 (3)	0.5717 (2)	0.0362 (6)	
H8	0.2563	0.4231	0.6470	0.043*	
C9	0.1903 (4)	0.1618 (3)	0.8844 (2)	0.0277 (5)	

### Atomic displacement parameters (Å^2^)

**Table:**

	*U*^11^	*U*^22^	*U*^33^	*U*^12^	*U*^13^	*U*^23^
Ni1	0.0261 (2)	0.0197 (2)	0.0281 (3)	−0.00600 (18)	−0.00623 (18)	−0.00112 (18)
S1	0.0318 (3)	0.0230 (3)	0.0241 (3)	−0.0083 (3)	−0.0059 (3)	0.0011 (2)
S2	0.0469 (4)	0.0278 (4)	0.0327 (4)	−0.0011 (3)	−0.0082 (3)	−0.0037 (3)
S3	0.0340 (4)	0.0228 (3)	0.0468 (4)	−0.0076 (3)	−0.0168 (3)	0.0003 (3)
N1	0.0434 (13)	0.0259 (12)	0.0269 (12)	−0.0008 (10)	−0.0041 (10)	−0.0013 (9)
N2	0.0330 (12)	0.0240 (11)	0.0307 (12)	−0.0052 (9)	−0.0015 (9)	−0.0023 (9)
N3	0.0765 (19)	0.0295 (13)	0.0277 (13)	−0.0020 (12)	0.0002 (12)	0.0000 (10)
N4	0.0385 (13)	0.0198 (11)	0.0445 (13)	−0.0046 (9)	−0.0188 (11)	0.0049 (9)
N5	0.0340 (12)	0.0276 (11)	0.0288 (11)	−0.0116 (9)	−0.0093 (9)	0.0012 (9)
N6	0.0429 (14)	0.0279 (12)	0.0567 (16)	−0.0054 (10)	−0.0258 (12)	0.0017 (11)
O1	0.0544 (12)	0.0267 (10)	0.0301 (10)	−0.0180 (9)	−0.0033 (9)	0.0025 (8)
O2	0.0408 (11)	0.0333 (10)	0.0267 (9)	−0.0107 (8)	−0.0032 (8)	−0.0040 (8)
O3	0.0351 (10)	0.0386 (11)	0.0395 (11)	−0.0074 (8)	−0.0133 (8)	0.0083 (9)
O4	0.0365 (11)	0.0407 (11)	0.0508 (12)	−0.0116 (9)	−0.0104 (9)	0.0009 (9)
C1	0.0329 (14)	0.0282 (14)	0.0292 (14)	−0.0086 (11)	−0.0039 (11)	−0.0020 (11)
C2	0.0365 (15)	0.0286 (14)	0.0281 (14)	−0.0054 (11)	−0.0067 (11)	0.0027 (11)
C3	0.0283 (13)	0.0273 (13)	0.0268 (13)	−0.0091 (10)	−0.0061 (10)	0.0018 (10)
C4	0.0261 (12)	0.0230 (13)	0.0275 (13)	−0.0077 (10)	−0.0075 (10)	0.0035 (10)
C5	0.0387 (15)	0.0277 (14)	0.0340 (15)	−0.0036 (12)	−0.0060 (12)	−0.0008 (11)
C6	0.0433 (16)	0.0236 (14)	0.0476 (18)	0.0003 (12)	−0.0104 (13)	0.0077 (12)
C7	0.0465 (17)	0.0413 (17)	0.0355 (16)	−0.0059 (14)	−0.0112 (13)	0.0119 (13)
C8	0.0419 (16)	0.0369 (16)	0.0256 (14)	−0.0058 (12)	−0.0038 (12)	0.0023 (11)
C9	0.0310 (14)	0.0263 (13)	0.0245 (13)	−0.0055 (11)	−0.0046 (11)	−0.0026 (10)

### Geometric parameters (Å, °)

**Table:**

Ni1—N5^i^	1.903 (2)	N5—H5A	0.9000
Ni1—N5	1.903 (2)	N5—H5B	0.9000
Ni1—S3^i^	2.1788 (7)	N6—C9	1.310 (3)
Ni1—S3	2.1788 (7)	N6—H6A	0.8600
S1—O1	1.4487 (18)	N6—H6B	0.8600
S1—O3	1.4590 (19)	O4—H4C	0.8500
S1—O2	1.4655 (18)	O4—H4D	0.8500
S1—C4	1.784 (2)	C2—C3	1.472 (3)
S2—C1	1.694 (3)	C2—H2	0.9300
S3—C9	1.720 (2)	C3—C8	1.400 (4)
N1—C1	1.340 (3)	C3—C4	1.400 (3)
N1—N2	1.377 (3)	C4—C5	1.390 (3)
N1—H1	0.8600	C5—C6	1.385 (4)
N2—C2	1.265 (3)	C5—H5	0.9300
N3—C1	1.319 (3)	C6—C7	1.378 (4)
N3—H3A	0.8600	C6—H6	0.9300
N3—H3B	0.8600	C7—C8	1.382 (4)
N4—C9	1.320 (3)	C7—H7	0.9300
N4—N5	1.423 (3)	C8—H8	0.9300
N4—H4	0.8600		
			
N5^i^—Ni1—N5	180.000 (1)	C9—N6—H6B	120.0
N5^i^—Ni1—S3^i^	88.41 (6)	H6A—N6—H6B	120.0
N5—Ni1—S3^i^	91.59 (6)	H4C—O4—H4D	108.2
N5^i^—Ni1—S3	91.59 (6)	N3—C1—N1	117.1 (2)
N5—Ni1—S3	88.41 (6)	N3—C1—S2	123.0 (2)
S3^i^—Ni1—S3	180.000 (1)	N1—C1—S2	119.80 (19)
O1—S1—O3	112.53 (11)	N2—C2—C3	120.2 (2)
O1—S1—O2	112.54 (11)	N2—C2—H2	119.9
O3—S1—O2	111.48 (11)	C3—C2—H2	119.9
O1—S1—C4	107.61 (11)	C8—C3—C4	118.1 (2)
O3—S1—C4	107.07 (11)	C8—C3—C2	119.0 (2)
O2—S1—C4	105.09 (11)	C4—C3—C2	123.0 (2)
C9—S3—Ni1	97.45 (9)	C5—C4—C3	120.6 (2)
C1—N1—N2	120.6 (2)	C5—C4—S1	117.17 (19)
C1—N1—H1	119.7	C3—C4—S1	122.23 (18)
N2—N1—H1	119.7	C6—C5—C4	120.1 (2)
C2—N2—N1	116.0 (2)	C6—C5—H5	119.9
C1—N3—H3A	120.0	C4—C5—H5	119.9
C1—N3—H3B	120.0	C7—C6—C5	119.9 (2)
H3A—N3—H3B	120.0	C7—C6—H6	120.0
C9—N4—N5	118.9 (2)	C5—C6—H6	120.0
C9—N4—H4	120.6	C6—C7—C8	120.3 (3)
N5—N4—H4	120.6	C6—C7—H7	119.8
N4—N5—Ni1	115.48 (15)	C8—C7—H7	119.8
N4—N5—H5A	108.4	C7—C8—C3	120.9 (3)
Ni1—N5—H5A	108.4	C7—C8—H8	119.5
N4—N5—H5B	108.4	C3—C8—H8	119.5
Ni1—N5—H5B	108.4	N6—C9—N4	119.8 (2)
H5A—N5—H5B	107.5	N6—C9—S3	121.3 (2)
C9—N6—H6A	120.0	N4—C9—S3	118.86 (19)
			
N5^i^—Ni1—S3—C9	174.91 (10)	O3—S1—C4—C5	111.1 (2)
N5—Ni1—S3—C9	−5.09 (10)	O2—S1—C4—C5	−7.5 (2)
C1—N1—N2—C2	−179.7 (2)	O1—S1—C4—C3	51.3 (2)
C9—N4—N5—Ni1	−10.8 (3)	O3—S1—C4—C3	−69.9 (2)
S3^i^—Ni1—N5—N4	−171.05 (16)	O2—S1—C4—C3	171.4 (2)
S3—Ni1—N5—N4	8.95 (16)	C3—C4—C5—C6	0.5 (4)
N2—N1—C1—N3	−4.5 (4)	S1—C4—C5—C6	179.4 (2)
N2—N1—C1—S2	176.70 (18)	C4—C5—C6—C7	0.3 (4)
N1—N2—C2—C3	178.2 (2)	C5—C6—C7—C8	−0.6 (5)
N2—C2—C3—C8	1.9 (4)	C6—C7—C8—C3	0.1 (4)
N2—C2—C3—C4	−177.2 (2)	C4—C3—C8—C7	0.7 (4)
C8—C3—C4—C5	−1.0 (4)	C2—C3—C8—C7	−178.4 (3)
C2—C3—C4—C5	178.1 (2)	N5—N4—C9—N6	−175.8 (2)
C8—C3—C4—S1	−179.8 (2)	N5—N4—C9—S3	5.8 (3)
C2—C3—C4—S1	−0.8 (3)	Ni1—S3—C9—N6	−177.3 (2)
O1—S1—C4—C5	−127.7 (2)	Ni1—S3—C9—N4	1.0 (2)

Symmetry codes: (i) −*x*+1, −*y*, −*z*+2.

### Hydrogen-bond geometry (Å, °)

**Table:**

*D*—H···*A*	*D*—H	H···*A*	*D*···*A*	*D*—H···*A*
N1—H1···S2^ii^	0.86	2.73	3.475 (2)	147
N3—H3A···N2	0.86	2.29	2.643 (3)	105
N3—H3A···O4^iii^	0.86	2.58	3.224 (3)	132
N4—H4···O4^iii^	0.86	1.97	2.794 (3)	160
O4—H4C···O2	0.85	1.97	2.819 (3)	172
O4—H4D···O3^iv^	0.85	2.19	3.036 (3)	172
O4—H4D···O4^v^	0.85	2.58	2.903 (3)	104
N5—H5A···O2^vi^	0.90	1.97	2.837 (3)	163
N5—H5B···O1^iii^	0.90	2.23	2.893 (3)	130
N6—H6A···O3^vii^	0.86	2.03	2.866 (3)	165
N6—H6B···S2^viii^	0.86	2.50	3.298 (2)	156
C2—H2···O1	0.93	2.50	3.066 (3)	119
C5—H5···O2	0.93	2.38	2.811 (3)	108
C8—H8···N2	0.93	2.48	2.790 (4)	100

Symmetry codes: (ii) −*x*, −*y*+2, −*z*+1; (iii) −*x*+1, −*y*+1, −*z*+1; (iv) −*x*+1, −*y*+1, −*z*; (v) −*x*+2, −*y*+1, −*z*; (vi) *x*, *y*, *z*+1; (vii) −*x*, −*y*+1, −*z*+1; (viii) *x*, *y*−1, *z*.
